# The Impact of Adult Vitamin D Deficiency on Behaviour and Brain Function in Male Sprague-Dawley Rats

**DOI:** 10.1371/journal.pone.0071593

**Published:** 2013-08-09

**Authors:** Jacqueline H. Byrne, Meggie Voogt, Karly M. Turner, Darryl W. Eyles, John J. McGrath, Thomas H. J. Burne

**Affiliations:** 1 Queensland Brain Institute, The University of Queensland, St Lucia, Queensland, Australia; 2 Queensland Centre for Mental Health Research, Wacol, Queensland, Australia; 3 Discipline of Psychiatry, The University of Queensland, St Lucia, Queensland, Australia; Chiba University Center for Forensic Mental Health, Japan

## Abstract

**Background:**

Vitamin D deficiency is common in the adult population, and this has been linked to depression and cognitive outcomes in clinical populations. The aim of this study was to investigate the effects of adult vitamin D (AVD) deficiency on behavioural tasks of relevance to neuropsychiatric disorders in male Sprague-Dawley rats.

**Methods:**

Ten-week old male Sprague-Dawley rats were fed a control or vitamin D deficient diet for 6 weeks prior to, and during behavioural testing. We first examined a range of behavioural domains including locomotion, exploration, anxiety, social behaviour, learned helplessness, sensorimotor gating, and nociception. We then assessed locomotor response to the psychomimetic drugs, amphetamine and MK-801. Attention and vigilance were assessed using the 5 choice serial reaction time task (5C-SRT) and the 5 choice continuous performance task (5C-CPT) and, in a separate cohort, working memory was assessed using the delay match to sample (DMTS) task. We also examined excitatory and inhibitory neurotransmitters in prefrontal cortex and striatum.

**Results:**

AVD-deficient rats were deficient in vitamin D_3_ (<10 nM) and had normal calcium and phosphate levels after 8–10 weeks on the diet. Overall, AVD deficiency was not associated with an altered phenotype across the range of behavioural domains tested. On the 5C-SRT AVD-deficient rats made more premature responses and more head entries during longer inter-trial intervals (ITI) than control rats. On the 5C-CPT AVD-deficient rats took longer to make false alarm (FA) responses than control rats. AVD-deficient rats had increases in baseline GABA levels and the ratio of DOPAC/HVA within the striatum.

**Conclusions:**

AVD-deficient rats exhibited no major impairments in any of the behavioural domains tested. Impairments in premature responses in AVD-deficient rats may indicate that these animals have specific alterations in striatal systems governing compulsive or reward-seeking behaviour.

## Introduction

Vitamin D deficiency is prevalent in the adult population [1] and there is a growing body of evidence showing that vitamin D has an impact on brain function [2], [3]. Several systematic reviews and meta-analyses have been undertaken, based on cross-sectional and prospective studies, that lend weight to hypotheses linking low vitamin D with (a) adverse cognitive outcomes and dementia [4], [5] and (b) depression [6].

Animal models can provide an efficient platform to explore clues from epidemiology and examine the biological plausibility of candidate exposures. The hypothesis that low vitamin D levels affect the developing brain is supported by data from prenatal vitamin D deficiency in rodents, in which the foetus develops in utero in a vitamin D-deficient but normocalcaemic dam [7], [8]. Prenatally vitamin D-deficient neonates had increased cell proliferation and reduced apoptosis [9]. Behaviourally, the adult offspring of prenatally vitamin D deficient dams exhibit transient “hyperlocomotion” [10]–[12], enhanced locomotor responses to psychomimetic drugs, such as MK-801 [13] and amphetamine [14], and impaired responses on cognitive tasks assessing latent inhibition [15] and response inhibition [16].

While there is now a considerable body of research examining the impact of developmental vitamin D (DVD) deficiency on brain outcomes, there is relatively little known about the impact of adult vitamin D (AVD) deficiency on rodent behaviour. One study based on male Sprague-Dawley rats, exposed the animals to a vitamin D-deficient diet from weaning [17]. After the prolonged exposure from early life, the rats had reduced body weight and musculoskeletal problems, which may have confounded the results [17]. However, the impact of AVD deficiency in normocalcaemic C57BL/6J and BALB/c adult mice has recently been assessed on a number of behavioural domains. The behavioural phenotype of AVD deficiency in mice was one of enhanced locomotion in a novel open field, but other features were dependent on the background strain; in addition AVD-deficient BALB/c mice spent more time on the open arms of an elevated plus maze, and had enhanced responses to aversive stimuli, including shock, heat and sound [18]. These findings are consistent with the behavioural effects seen after pharmacological modulation of GABAergic neurotransmission [19], [20]. Interestingly, AVD-deficient BALB/c mice had significantly higher levels of GABA and glycine, as well as lower levels of glutamate and glutamine, in whole brain tissue [18].

The overall goal of this study was to examine AVD deficiency in normocalcaemic adult male Sprague-Dawley rats. We selected adult rats (10 weeks of age) and maintained them on a diet free from vitamin D but with normal calcium levels for 6 weeks to induce vitamin D deficiency. The first aim was to assess behavioural domains of relevance to neuropsychiatric disorders, including locomotion, anxiety-related behaviour, learned helplessness, exploration, sensorimotor gating, social behaviour and psychomimetic-induced locomotion. We also assessed the cognitive domains of attention and working memory using the 5 choice serial reaction time task (5C-SRT), 5 choice continuous performance task (5C-CPT) and delay match to sample (DMTS) [36]. The 5C-SRT and 5C-CPT are used to explore conditional associative learning, selective attention, impulsivity and motivation [35], [37], [38]; whereas, the DMTS task investigates spatial working memory [39]. Finally, we screened for changes in excitatory and inhibitory neurotransmitters in selected brain regions (prefrontal cortex (PFC) and striatum). The PFC has long been implicated in cognition, including the aspects to be assessed in this study, operant learning, attention and working memory [21]. Based on both previous neurochemical changes found in AVD-deficient BALB/c mice [18] and the brain structures that are involved in cognitive functioning, we measured neurotransmitter levels that are potentially compromised in AVD-deficient rats, in the PFC and striatum.

## Results

### Home Cage Behaviour

There was no significant effect of diet on the rats’ initial habituation to the PhenoTyper cages. Over three subsequent days both test groups showed normal 24-hour circadian rhythms (i.e. greater activity during the dark compared to the light phase of the cycle). There was no overall significant main effect of diet on activity levels across the day (*F*_(1,28)_ = 0.51, *p* = 0.47), data not shown.

### Behavioural Test Battery

The overall phenotype of the AVD-deficient rats was similar to control rats across a range of behavioural domains. A brief description of the results on each of the behavioural tests is given below and a summary of the results, sorted by behavioural domain is displayed in Table 1.

**Table 1:** Mean ± S.E.M. values from the behavioural test battery showing the behavioural domains of control and AVD-deficient rats.

Domain	Test	Parameter Measured	Control	*n*	AVD-deficient	*n*
Body weight	Start of experiment	**Body weight (g)**	346.80±59.90	20	341.68±52.37	20
	End of experiment	**Body weight (g)**	508.84±52.25	20	501.70±45.01	20
Anxiety	Elevated plus maze	**Duration on open arms (%)**	20.75±1.98	20	17.76±2.69	20
	Light-dark	**Latency for head emergence (s)**	13.42±1.43	19	10.22±1.02	20
Locomotion	Elevated plus maze	**Distance travelled (cm/10min)**	3001.06±213.55	20	2747.38±185.84	20
	Holeboard	**Distance travelled (cm/10min)**	3608.04±183.27	20	3585.35±191.76	20
	Open field	**Horizontal activity (cm/60 min)**	1693.88±247.18	20	1977.87±225.32	20
Exploration	Holeboard	**Head-dipping (counts/10 min)**	16.25±1.5	19	15.75±2.06	20
	Holeboard	**Rearing (counts/10 min)**	36.5±2.51	19	30.7±3.27	20
	Open field	**Vertical (counts/10 min)**	34.95±5.80	20	37.05±3.62	20
Social Behaviour	Social interaction	**Latency to investigate (s)**	100.76±16.19	10	110.09±16.50	10
	Social interaction	**Investigation (counts/10 min)**	83.95±6.25	10	87.00±6.72	10
Aggression	Social interaction	**Aggression (counts/10 min)**	0.60±0.45	10	1.65±0.75	10
Learned helplessness	Forced swim test Day 1	**Immobile time (%)**	31.46±2.66	20	29.34±2.40	20
	Forced swim test Day 2	**Immobile time (%)**	17.76±2.68	20	23.89±3.26	20
Avoidance learning	Active avoidance	**Conditioned avoidance response (%)**	27.08±5.14	17	30.19±5.77	18
Nociception	Tailflick	**Latency to flick (s)**	4.98±0.25	20	5.57±0.87	20
	Hotplate	**Latency to lick hindpaw (s)**	11.75±0.59	20	12.04±0.94	20

#### EPM

All rats entered the open arms of the EPM and the time spent in the closed arms was preferred by both control (*t*_(19)_ = 2.30, *p* = 0.03) and AVD-deficient rats (*t*_(19)_ = 3.17, *p* = 0.005). There were no significant differences between control and AVD-deficient rats on the distance travelled (*t*_(38)_ = 0.90, *p* = 0.38), frequency (*t*_(38)_ = 0.75, *p* = 0.46) or duration (*t*_(38)_ = 0.89, *p* = 0.38) on the open arms of the EPM.

#### Holeboard test

All rats explored the holeboard by poking their head into the holes. There were no significant differences between control and AVD-deficient rats on distance travelled, latency of head-dipping (*t*_(38)_ = 0.45, *p* = 0.65), frequency of head-dipping (*t*_(38)_ = 0.20, *p* = 0.85) or frequency of rearing (*t*_(38)_ = 1.41, *p* = 0.17) during the holeboard test.

#### Light-dark test

The AVD-deficient rats had a shorter latency for head emergence on the light-dark test compared to controls, but this failed to reach significance (*t*_(37)_ = 1.84, *p* = 0.07). There was no significant effect of diet on latency for body emergence (*t*_(37)_ = 1.62, *p* = 0.11) or frequency of crossings (*t*_(37)_ = 0.06, *p* = 0.95).

#### Social interaction test

There were no significant differences between control and AVD-deficient rats on the latency of investigation behaviour (*t*_(18)_ = 4.03 *p* = 0.69) or frequency (*t*_(18)_ = 0.33, *p* = 0.74). There were also no differences between groups in the frequency of rearing (*t*_(18)_ = 1.31, *p* = 0.21), self-grooming (*t*_(18)_ = 2.38, *p* = 0.12) or aggressive behaviour (*t*_(18)_ = 1.20, *p* = 0.25).

#### Two-day forced swim test

There was no significant main effect of diet on time spent immobile on day 2 of the forced swim test (*F*_(1,37)_ = 1.92, *p* = 0.18). There was also no effect of testing order (*F*_(1,36)_ = 0.00, *p* = 0.96) or time spent immobile on day 1 (*F*_(1,36)_ = 0.36, *p* = 0.55). All rats showed a significant increase in the time spent immobile over time on day one (*t*_(39)_ = 11.45, *p*<0.01) and a similar response was seen on day 2 (*t*_(38)_ = 7.29, *p*<0.01).

#### Nociception: Tail flick and hot-plate test

Control and AVD-deficient rats showed similar latencies to respond on the tail flick (*t*_(38)_ = 0.65, *p* = 0.52) and hot-plate tests (*t*_(38)_ = 0.26, *p* = 0.79).

#### Prepulse inhibition (PPI) of acoustic startle response (ASR)

**Figure 1:**
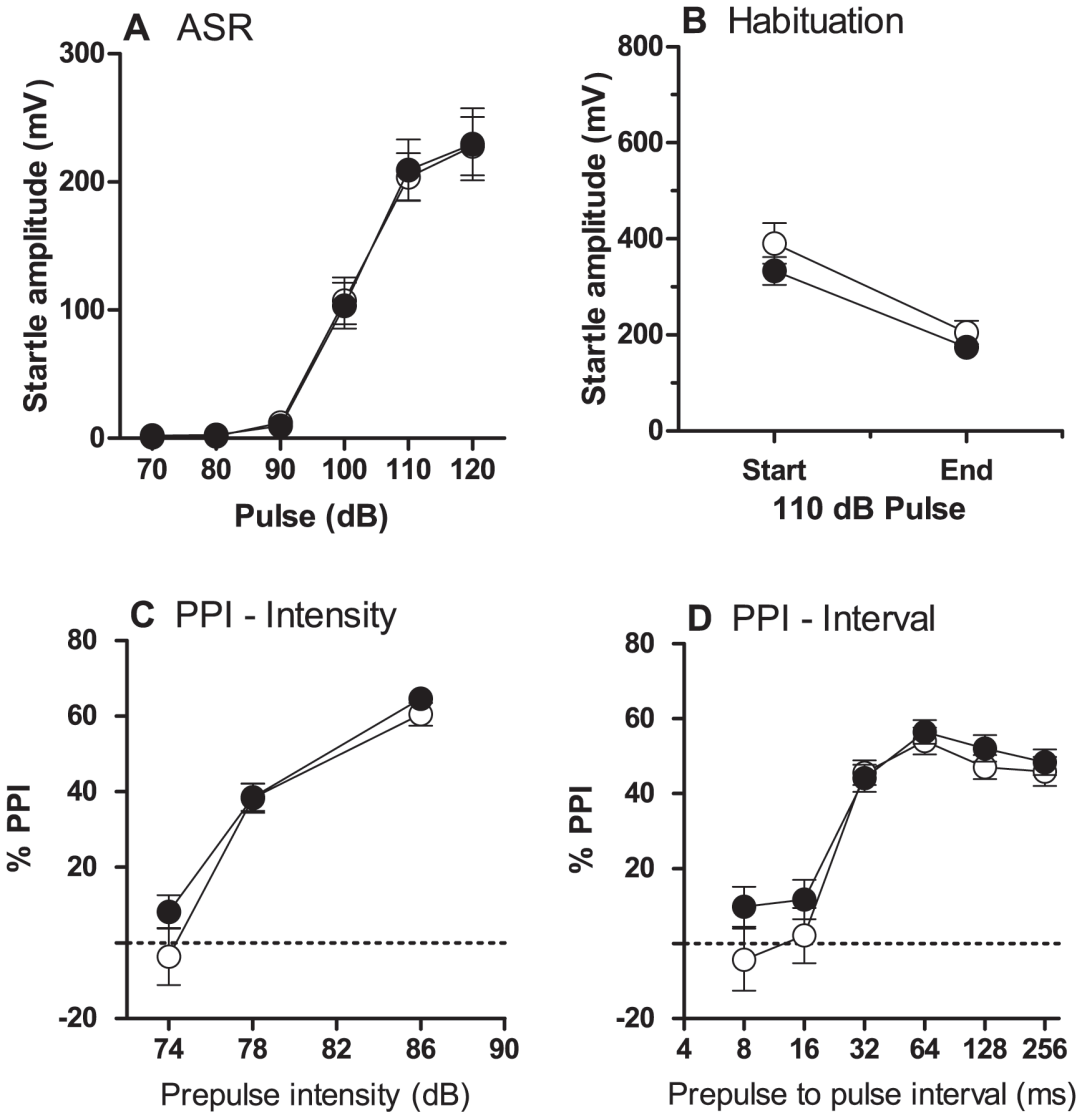
Performance of rats on the prepulse inhibition (PPI) of the acoustic startle response (ASR). This figure shows the ASR (A), within session habituation (B), PPI based on prepulse intensity (C) and PPI based on prepulse to pulse interval (D) for control (n = 34, ○) and AVD-deficient (n = 34, •) rats over the five different inter-trial intervals ranging 3–7 s. Data were analysed using repeated measures ANOVA and are expressed as mean ± SEM.

All rats responded to increasing pulse amplitude with increased startle response. Overall, there was no significant effect of diet on ASR and within-session habituation was seen in both control and AVD-deficient rats. There was no significant main effect of diet on PPI scores (Fig. 1).

#### Active avoidance

Both test groups progressively learned to avoid the footshock (*F*_(1,33)_ = 55.1, *p*<0.001). No significant effect of AVD deficiency was found on %CAR (*F*_(1,33)_ = 1.46, *p* = 0.71) and there was no difference in the latency to avoid the footshock over 80 trials (*F*_(1,33)_ = 1.62, *p* = 0.21).

#### Open field test

Both test groups showed similar habituation responses to the open field test. At T30, AVD-deficient rats had significantly increased horizontal activity (*t*_(38)_ =  = 2.46, *p* = 0.02) and vertical counts (*t*_(38)_ = 2.06, *p* = 0.05). There was no significant main effect of AVD deficiency on horizontal activity (*F*_(1,38)_ = 0.72, *p* = 0.40) or vertical counts (*F*_(1,38)_ = 0.09, *p* = 0.76) in the open field test.

### Behavioural Pharmacology

**Figure 2:**
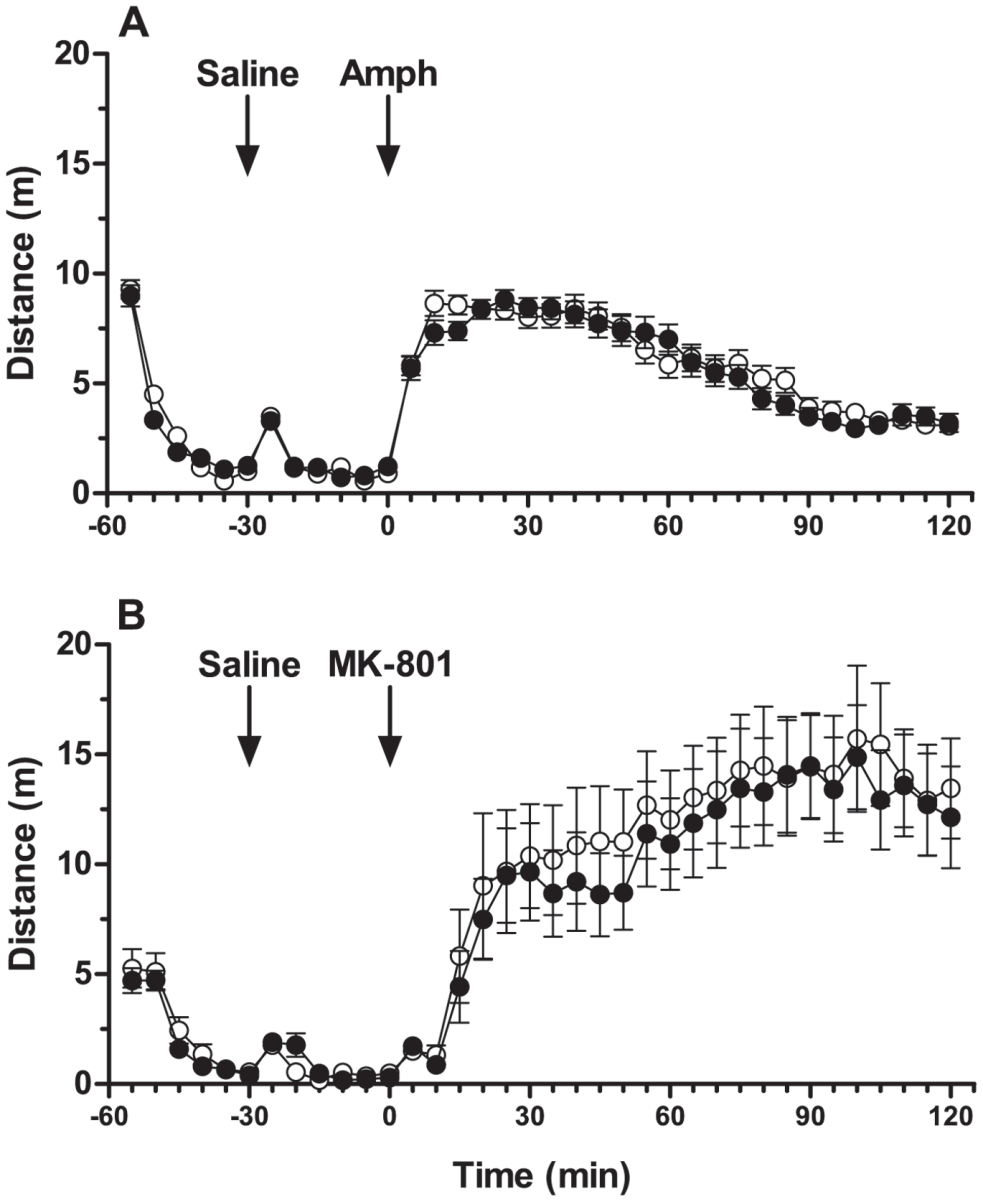
Performance of rats after treatment with the psychomimetic drugs amphetamine (A) and MK-801 (B). Rats were habituated to the activity monitors for 30 min, received a saline injection and then 30 min later were given either 1.25 mg/kg d-amphetamine for control (n = 34, ○) and AVD-deficient (n = 34, •) or 0.5 mg/kg MK-801 for control (n = 21, ○) and AVD-deficient (n = 21, •). Data were analysed using repeated measures ANOVA and are expressed as mean ± SEM.

Control and AVD-deficient rats showed the same habituation response to a novel environment. Both groups of rats had a similar response to saline injection and showed a significant increase in the peak of horizontal activity 5 min after injection (*t*_(41)_ = 7.32, *p*<0.001). Both test groups showed a characteristic response to amphetamine (Fig. 2A) and to MK-801 (Fig. 2B) and there was no significant effect of diet.

### Operant Behaviours

#### 5C-SRT

**Figure 3:**
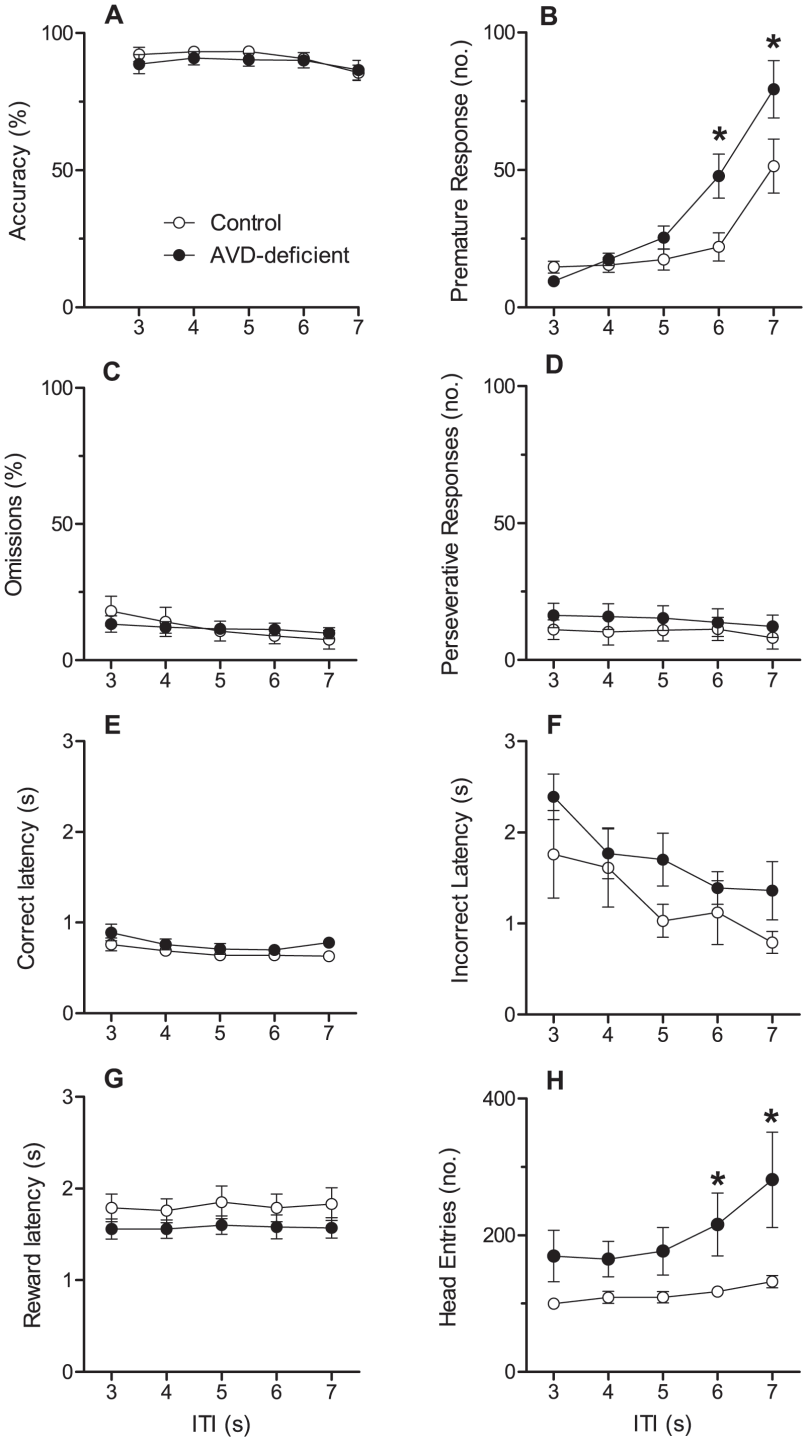
Performance of rats on the 5C-SRT with multiple inter-trial intervals. This figure shows accuracy (A), premature responses (B), the omission rate (C), perseverative responses (D), correct latency (E), incorrect latency (F), reward latency (G) and head entries (H) for control (n = 7, ○) and AVD-deficient (n = 7, •) rats over the five different inter-trial intervals ranging 3–7 s. Data were analysed using repeated measures ANOVA and are expressed as mean ± SEM. *p<0.05 between control and AVD-deficient rats, *t*-test.

There was no effect of diet on the time taken to progress though the different levels of training (*t_(13)_* = 0.08, *p* = 0.93). One rat from each dietary group was excluded for not reaching baseline criteria, leaving a final sample size of n = 7 per diet. There was a significant effect of ITI on performance on the 5C-SRT (accuracy, omissions, premature response, head entries, and correct and incorrect latencies, *F_(4,48)_* >5.0, *p*<0.05, Fig. 3). There was no significant effect of diet on accuracy or number of omissions (*F_(4,48)_* = 0.29, *p* = 0.6 and *F_(4,48)_* = 0.01, *p* = 0.96, respectively); furthermore accuracy remained over 85% regardless of diet and ITI. There was no significant diet by ITI interaction on accuracy or omissions (*F_(4,48)_* = 0.82, *p* = 0.52 and *F_(4,48)_* = 2.22, *p* = 0.08, respectively).

There was no significant main effect of diet on any of the three latency measures (correct latency, incorrect latency, or reward latency *F_(4,48)_* <2.0, *p*>0.05) and no diet by ITI interaction on any latency (correct latency; incorrect latency; or reward latency *F_(4,48)_* <2.0, *p*>0.05).

There was a significant effect of ITI on the number of premature responses, with rats making more premature responses in trials with a longer ITI (*F_(4,48)_* = 43.46, *p*<0.001). Moreover, there was a significant diet by ITI interaction on the number of premature responses, with AVD-deficient rats making more premature responses on longer ITI trials than controls (*F_(4,48)_* = 5.02, *p* = 0.002). However, the main effect of diet on the number of premature responses across all trials did not reach significance (*F_(4,48)_* = 3.88, *p* = 0.07).

All rats made significantly more head entries into the food magazine during the longer ITI (*F_(4,48)_* = 10.86, *p*<0.001). Again there was a significant diet by ITI interaction, with AVD-deficient rats making more head entries during the longer ITI (*F_(4,48)_* = 4.17, *p* = 0.006). There was no significant effect of ITI, diet, or any diet by ITI interaction on perservative responses (*F_(4,48)_* <2.0, *p*>0.05).

#### 5C-CPT

**Figure 4:**
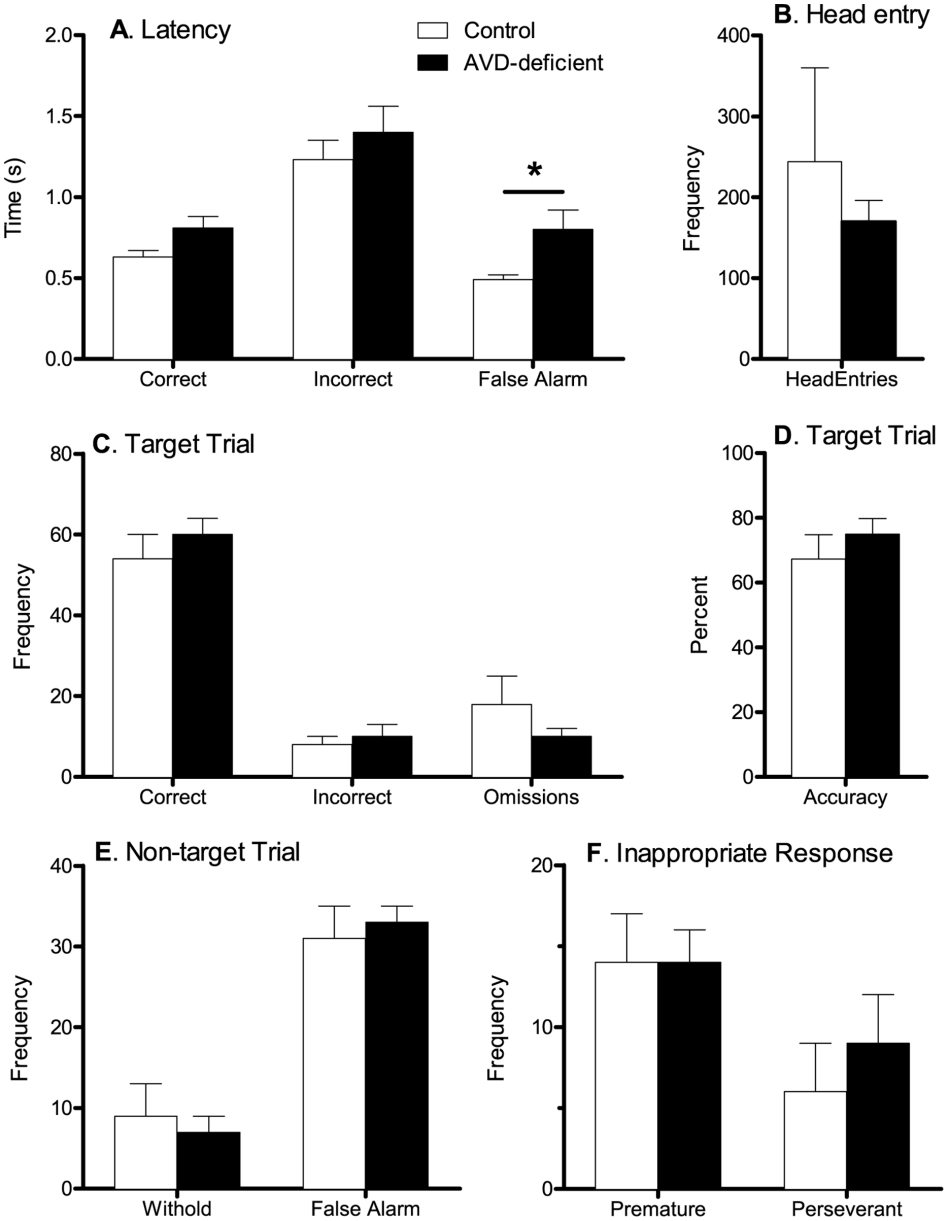
Performance of rats on the 5C-CPT. This figure shows the latency (A) head entries (B), response on target trials (C and D), non-target trials (E) and premature and perseverant responses (F) for control (n = 7) and AVD-deficient (n = 7) rats. Data were analysed using repeated measures ANOVA and are expressed as mean ± SEM. *p<0.05 between control and DVD-deficient rats, *t*-test.

Over the 10 5C-CPT sessions rats made more correct than incorrect responses and a difference was discovered in the response latencies, with correct responses being made much faster than incorrect responses. False alarm (FA) responses were also made quicker than incorrect responses (Fig. 4). There was no main effect of diet on the majority of the variables measured in the 5C-CPT. There was no effect of diet on the number of food rewards achieved or the number of omissions on target trials (*t_(11)_* = 0.89, *p* = 0.39 and *t_(11)_* = 1.21,*p* = 0.25, respectively). There was also no significant effect of diet on the number of head entries made in the target trials (*t_(11)_* = 0.68, *p* = 0.65). In the non-target trials it was predicted that AVD-deficient rats (n = 7) would be more impulsive and make more FA responses than control rats (n = 6), however this was not significant (*t_(11)_* = 0.60, *p* = 0.56). Interestingly, there was a main effect of diet on FA latency in the non-target trials (*t_(11)_* = 2.25, *p* = 0.046). AVD-deficient rats took longer to make a FA response (mean = 0.78±0.12 ms) than control rats (mean = 0.49±0.03 ms). No effect of diet was found on overall response latency (*t_(11)_* = 1.97, *p* = 0.08) and in the target trials there was no main effect of diet on correct response latency (*t_(11)_* = 2.00, *p* = 0.07). The diet by ITI interaction that was discovered in the 5C-SRT was followed up; however, there was no significant effect of diet on the proportion of premature responses in the 5C-CPT regardless of ITI (ITI 6 s: *t_(11)_* = 0.95, *p* = 0.36 and ITI 7 s: *t_(11)_* = 0.51, *p* = 0.62).

#### DMTS

**Figure 5:**
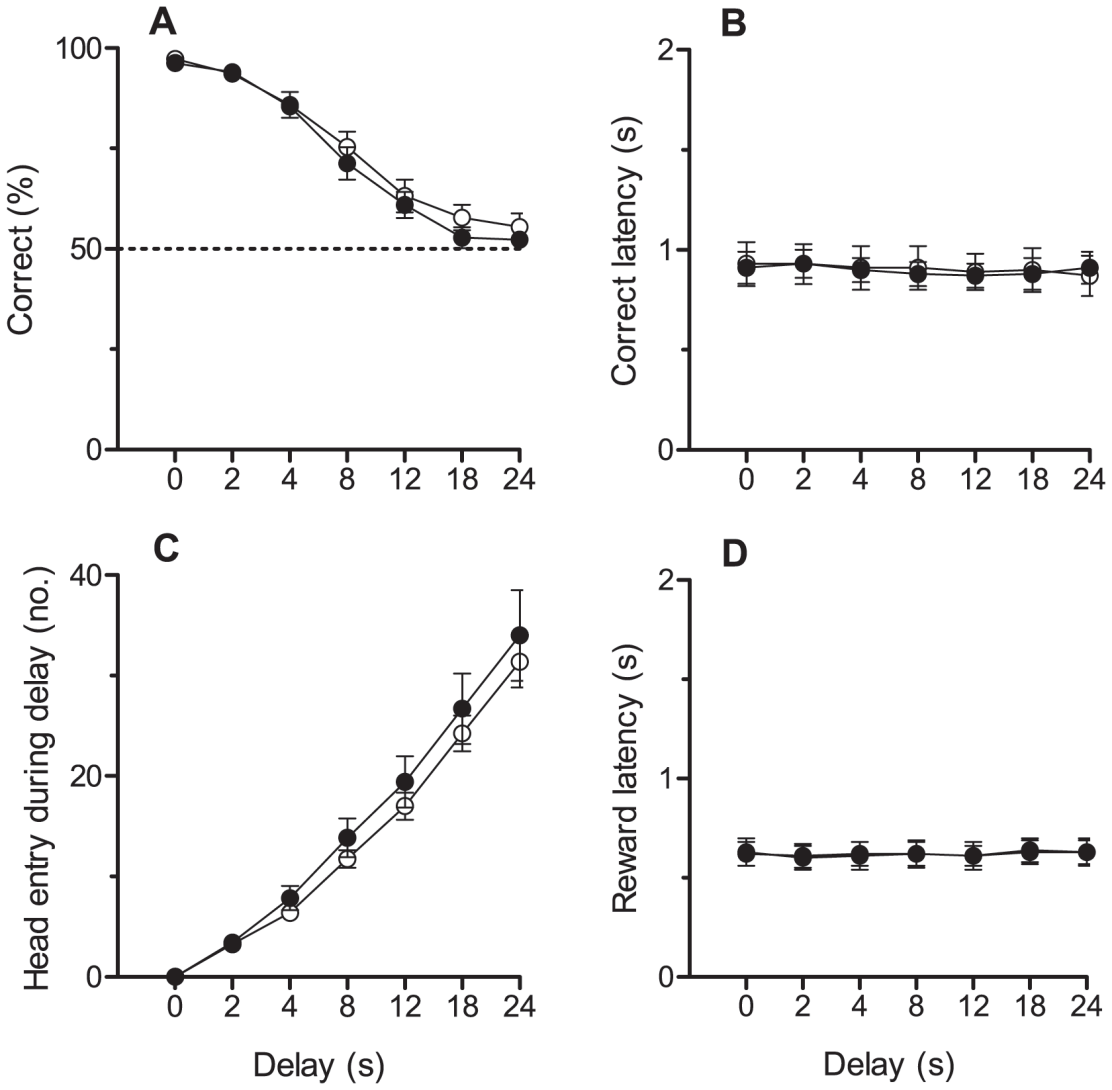
Performance of rats on the DMTS. This figure shows the percent correct (A), correct latency (B), head entries during delay (C) and reward latency (D) for control (n = 8, ○) and AVD-deficient (n = 7, •) rats. Data were analysed using repeated measures ANOVA and are expressed as mean ± SEM.

There was no significant effect of diet on the time taken to reach criteria for DMTS. One animal was excluded for not reaching criteria, leaving a final sample size of AVD-deficient rats n = 7 and control rats n = 8. No main effect of diet was found on the number of rewards received (*t_(13)_* = 0.77, *p* = 0.46) or the number of omissions (*t_(13)_* = 0.80, *p* = 0.44). There was no significant effect of diet for any parameter measured on DMTS (head entry, correct latency, incorrect latency, head entry latency, Fig. 5). When the delay period was greater than 12 s, the performance of both groups decreased, and it was during these longer delay periods that rats made more head entries and head entry latency increased in proportion to delay duration.

### Neurochemistry

There was no significant effect of diet on body weight (*t_(13)_* = 0.15, *p* = 0.88, Table 1) or brain weight (*t_(13)_* = 0.77, *p* = 0.45). There was a significant main effect of diet on GABA levels in the striatum (*t_(14)_* = 2.21, *p* = 0.05), with AVD-deficient rats (mean = 2313.51±97.25 pg/mg) having higher baseline levels of GABA than control rats (mean = 2019.75±91.10 pg/mg). There was a significant effect of diet on the DOPAC/HVA ratio in the striatum whereby AVD-deficient rats (mean = 3.17±0.15 pg/mg) had a higher DOPAC/HVA ratio than controls (mean = 2.72±0.11 pg/mg; *t_(14)_* = 2.14, *p* = 0.03), reflecting decreased conversion of DOPAC to HVA. No significant differences were found for any other neurotransmitter or metabolite examined (Table 2).

**Table 2:** Mean ± SEM values for brain neurochemistry.

PFC (pg/mg)	Control		AVD-Deficient	
NA	144.8	±13.3	134.1	±23.8
DOPAC	119.0	±14.3	104.2	±11.4
DA	22.4	±3.0	21.5	±4.0
5HIAA	398.4	±35.9	328.6	±25.4
HVA	116.4	±8.4	89.0	±10.7
5HT	46.6	±3.8	47.5	±6.8
Glu	8220.1	±536.0	7404.5	±350.73
GABA	1888.5	±133.3	1692.4	±77.1
DOPAC/DA	5.8	±0.8	5.8	±1.2
HVA/DA	5.9	±1.0	5.2	±1.0
DOPAC/HVA	1.0	±0.1	1.2	±0.2
5HIAA/5HT	8.8	±0.9	7.8	±1.2
DA/NA	0.1	±0.1	0.1	±0.0
NA/DA	7.0	±0.7	7.1	±1.1
**Striatum (pg/mg)**				
NA	587.1	±88.7	771.0	±85.3
DOPAC	3117.8	±93.8	3397.5	±155.54
DA	2294.8	±239.7	1853.1	±161.32
5HIAA	383.8	±21.2	395.7	±24.0
HVA	1153.0	±40.5	1073.1	±15.9
5HT	51.9	±5.4	54.3	±8.0
Glu	6450.9	±372.3	5722.1	±501.36
GABA*	2019.7	±91.1	2313.5	±97.2
DOPAC/DA	1.5	±0.2	1.9	±0.2
HVA/DA	0.5	±0.1	0.6	±0.1
DOPAC/HVA*	2.7	±0.1	3.1	±0.1
5HIAA/5HT	7.8	±0.8	8.0	±0.8
DA/NA	4.8	±1.0	2.7	±0.5
NA/DA	0.3	±0.1	0.4	±0.0

Abbreviations: NA, noradrenaline, DOPAC, 3,4 dihydroxyphenylacetic acid, DA, dopamine, 5HIAA, 5-hydroxyindolacetic acid, HVA, homovanillic acid, 5HT, 5-hydroxytryptamine, Glu, glutamate, GABA, γ-aminobutyric acid, *p<0.05.

## Discussion

Overall, our results clearly demonstrate that adult vitamin D (AVD) deficiency in male Sprague-Dawley rats was not associated with a gross neurochemical or behavioural phenotype. However, subtle changes on selected aspects of cognitive processing and on neurotransmitter signalling in the striatum were found. In the 5C-SRT, AVD-deficient rats showed more premature responses and a greater number of head entries in the longer ITI, and an increase in FA latency was found on the non-target trials of the 5C-CPT. In addition, the AVD-deficient rats had changes in the neurotransmitter content of the striatum, with an overall increase in GABA and an increased DOPAC/HVA ratio. These results suggest that, although subtle, AVD deficiency in the rat is associated with specific alterations in brain neurochemistry and behaviour.

AVD deficiency had no overall effect on locomotion and 24-hour circadian rhythm in their home cage. The AVD-deficient rats displayed normal responses during the remaining behavioural test battery, which included measures of locomotion, anxiety, exploration, social interaction, learned helplessness and avoidance learning. Nociceptive testing confirmed normal neural signalling in response to noxious stimuli. Thus, the results from the active avoidance paradigm were not confounded by differences in pain threshold. In contrast to behavioural features identified in rats exposed to developmental vitamin D deficiency, AVD deficiency was not associated with altered responses to MK-801 [13] or amphetamine [14].

Both groups completed training on the 5C-SRT at the same pace, performed the task with greater than 85% accuracy and no difference was found on the number of omissions. This confirms the AVD-deficient rats did not have any learning or attentional deficits. There was no difference in reward latency, which indicates both groups were equally motivated to perform the task and that motoric responses were normal, further verifying there were no musculoskeletal problems. However, AVD-deficient rats showed an increase in the number of premature responses and head entries for the longer ITI (6 and 7s). This implies an impulsive phenotype, in that AVD-deficient rats had difficulty withholding responses until presentation of the stimulus. Surprisingly, when these parameters were explored in the 5C-CPT, there was no significant effect of diet on the proportion of premature responses or the number of head entries made. Furthermore, there was no difference in the number of FA responses made in the non-target trials by AVD-deficient rats. A recent study has shown that DVD-deficient rats performed with comparable accuracy to controls on most measures of vigilance on target trials, however they exhibited impulsivity, or a lack of inhibition on non-target trials on the 5C-CPT [16]. These rodents also made more premature responses within the 5C-CPT paradigm. It may be that AVD-deficient rats showed reduced inhibitory control early-on during testing (during the 5C-SRT) but this diminished with further response inhibition training and so was absent during the 5C-CPT. By contrast DVD-deficient rats show deficits with inhibitory control under the additional load of the 5C-CPT [16]. Although there was no difference in the number of FA responses made, a significant increase in the latency to make a FA response was found for AVD-deficient rats. There were no effects of diet on any other response latency (correct, incorrect or reward) and so it is unlikely that there were differences in motivation.

There was no effect of diet on any DMTS parameter, which suggests that AVD-deficient rats did not have spatial working memory deficits. A clear decay curve was depicted, which showed all rats performing with high accuracy (<90%) in the short delays (0 and 2 s) and at chance (∼ 50%) after a delay period of more than 12 s. This further verifies the utility of the DMTS protocol for assessing working memory. The number of head entries and the corresponding latency increased in proportion to delay duration, indicating sustained attention to the task. Other latencies were held constant with minimal variability across delay duration and diet, confirming all rats’ performed at the same level.

AVD-deficient rats had increased GABA levels in the striatum. Tenenhouse [22] assessed neurotransmitter content in chronic vitamin D-deficient rats and found increased GABA in most regions investigated. These rats were hypocalcaemic and no differences in GABA were discovered when calcium levels were normalised [22]. Recently, elevated levels of GABA have been found in whole brain from AVD-deficient BALB/c mice [18]. Furthermore, addition of 1,25-(OH)2D3 can ameliorate the toxic effects of neurotoxic agent 6-hydroxydopamine [23]. This possibly occurs via recruitment of neurotrophic agents, for example GDNF [24], and 6-hydroxydopamine has also been associated with increased GABA in the striatum [25]. One study explored the role of striatal GABA in a sequential learning task and found that learning of new sequences was impaired after a GABA agonist was injected into the anterior caudate and the putamen; whereas, the execution of well-learned sequences was disrupted when the GABA agonist was injected into the middle-posterior putamen and, although less severely, also the anterior caudate and putamen [26]. Future research should investigate the potential role of vitamin D-deficiency on GABA, and possible implications on cognition.

The ratio of DOPAC/HVA was significantly higher in the striatum of AVD-deficient rats, indicating that less DOPAC was metabolised to HVA. DVD-deficient neonates also had reduced conversion of DOPAC to HVA. This was associated with a 45% reduction in the expression of catechol-O-methyl transferase (COMT), the enzyme involved in the conversion of DOPAC to HVA, however no other changes in neurochemistry or MAO expression (the enzyme involved in converting DA to DOPAC were found [27]. An increased DOPAC/HVA ratio is consistent with a reduction in COMT gene expression, even with normal levels of DOPAC. A COMT-deficient mouse model had normal DA, elevated DOPAC although no HVA [28]. Early postnatal vitamin D supplementation increased HVA level in the adult forebrain [29]. Disruption to striatal dopamine function is typically associated with altered behaviours, such as hyperactivity, impaired sensorimotor gating and reduced cognitive flexibility [30], which we did not see in the current study. Impulsive behaviours have been associated with monoaminergic dysfunction in the striatum [31] and it is possible that the increased DOPAC/HVA ratio contributed to the mild impulsivity response observed in the 5C-SRT in the current study.

There are several limitations with the model of AVD deficiency used in this study and further refinements may yield more striking results. Exploring a longer period of vitamin D deficiency, or subsequent insults may have a greater impact on brain function and behaviour. AVD-deficient rats had lower blood serum 25-hydroxyvitamin D than controls, future studies should investigate the effects of longer periods of insufficiency or deficiency.

Despite rodents having a nocturnal lifestyle, shaved rat skin is able to synthesise vitamin D after UVB exposure [32], and hair-coated animals, such as dairy cows, synthesise vitamin D_3_ [33], suggesting that a full coat of hair does not prevent cutaneous vitamin D production. Like humans, rodents also gain vitamin D from the diet but the main difference between rodents and humans is the bioavailability of vitamin D_2_ and D_3_ [34]. In our experiments we have only manipulated vitamin D_3_ levels, and the diets do not contain any vitamin D_2_ and we could not detect any vitamin D_2_ in blood samples from the rats used in the current study. The lack of a marked behavioural phenotype in the AVD model in the male Sprague-Dawley rat may have important implications for future animal models exploring the biological mechanisms that may underpin the epidemiological associations between low vitamin D and adverse brain outcomes. While low vitamin D in the adult rodent is characterised by several behavioural and neurochemical changes in the BALB/c mouse [18], the phenotype of AVD in the Sprague-Dawley rat is essentially normal.

Thus, we recommend that researchers consider this species for future research models. Recently, evidence has emerged from animal models to suggest that vitamin D insufficiency or deficiency may exacerbate the progression of an underlying brain disorder. For example, in a study of ischemia it was reported that animals allocated to a vitamin D deficient diet (prior to the stroke lesion) subsequently had significantly greater ischemic brain damage and worse functional impairments, compared to rodents on a vitamin D replete diet [35]. This finding is in keeping with the well-known neuroprotective properties of vitamin D [3]. For example, vitamin D is a potent inducer of nerve growth factor (NGF) [36], [37]. In addition, vitamin D has a direct neuroprotective action against excitotoxic insults by down regulating L-type calcium channels [38] and pre-treatment with vitamin D attenuates the effects of various stressors including 6-hydroxydopamine-induced neurotoxicity [39]. Thus, we speculate that the epidemiological clues linking low vitamin D and adverse neurocognitive outcomes may be mediated by ‘two hits’ – in other words low vitamin D may exacerbate a separate neurobiological stressor. Evidence from a recently published randomized controlled trial of vitamin D supplementation for the treatment of Parkinson’s Disease (PD) support this hypothesis [40]. This study found that those on placebo (and thus, those more likely to have persisting 25 OHD insufficiency or deficiency), had a steady worsening on PD outcomes. In contrast, those on vitamin D supplements had no change in PD outcomes over the year. The results strongly suggest that low vitamin D status exacerbates disease progression [41].

The main finding of this study is that AVD-deficient rats had, overall, a normal behavioural and neurochemical phenotype. However, there were specific aspects of attentional processing (premature responses on 5C-SRT and false alarm latency on 5C-CPT) that were affected by AVD deficiency. The behavioural findings may be associated with increases in baseline GABA and an altered ratio of DOPAC/HVA within the striatum, and these should be targets for future experiments. Further refinements to the model, such as combined with second hit exposures, may be necessary to more adequately model the effects of vitamin D deficiency in human populations.

## Materials and Methods

### Ethics Statement

All procedures were performed with the approval of The University of Queensland Animal Ethics Committee, under the guidelines of the National Health and Medical Research Council of Australia.

### Animals and Housing

A total of 213 outbred male Sprague-Dawley rats (Australian Institute for Bioengineering & Nanotechnology, Brisbane) were used. Rats were housed in pairs in macrolon cages with aspen bedding and blocks (P.J. Murphy Forest Products Corp., NJ, USA). The rats were maintained on a 12-hour light-dark cycle schedule (lights on at 07∶00 h) with *ad libitum* access to water and food (Speciality Feeds, WA, Australia). Ten-week old rats were assigned pseudorandomly to either a control (AIN93G with 1,000 I.U. vitamin D_3_) or vitamin D_3_ deficient diet (AIN93G with 0 I.U. vitamin D_3_) for a minimum of 6 weeks prior to and during testing procedures. Body weight was measured each week throughout the experiment.

### Blood Sera

Dietary vitamin D_3_ intake is strongly associated with serum levels of 25-hydroxyvitamin D_3_ in rats [42]. At the end of experimentation, a terminal blood sample was taken from each rat by cardiac puncture. The levels of 25-hydroxyvitamin D_2_ and D_3_ were determined from sera samples (Control *n* = 58, AVD *n* = 60) using liquid chromatography-tandem mass spectrometry (Sciex Instruments, ON, Canada) on a 4000 QTrap API AB mass spectrometer [43], [44]. Serum calcium (Ca^2+^) and phosphate (PO_4_^3−^) levels were determined from a representative population (Control *n* = 15, AVD *n* = 15) using a commercial supplier (Queensland Health Pathology and Scientific Services, Princess Alexandra Hospital, Brisbane, Australia). Despite variation in serum 25-OHD_3_ concentrations, all rats within the AVD-deficient group had lower vitamin D_3_ levels than controls (Control: 60.0±2.0 nM, AVD-deficient: 8.7±0.7 nM, *t*_(116)_ = 24.59, *p<*0.001), while maintaining normal Ca^2+^ (Control: 2.44±0.13 mM, AVD-deficient: 2.48±0.12 mM, *t*_(28)_ = 0.90, *p = *0.73) and PO_4_^3−^ (Control: 2.84±0.42 mM, AVD-deficient: 2.82±0.44 mM, *t*_(28)_ = 0.90, *p = *0.73) levels. Vitamin D_2_ levels were undetectable in all samples.

### Home Cage Behaviour

Thirty rats were used to assess home cage behaviour (Control *n* = 14, AVD *n* = 16). Four PhenoTyper boxes (Noldus, Wageningen, The Netherlands) were modified by the addition of a clear plastic partition to permit testing of two individually housed rats per box (to test eight rats simultaneously). Activity was monitored using an inbuilt overhead camera sensitive to infrared light, attached to automated video tracking software (EthoVision XT, Noldus, Wageningen, The Netherlands). The software recorded the spatial location of the centre of mass for each rat every second and calculated the distance the rat moved per unit time. A video image was obtained regardless of visible light levels in the room, because the cage was illuminated via infrared lights situated within the lid of the cage. Activity was monitored over a continuous 90-hour period. Habituation to the cages was deemed as 300 min of initial recorded activity.

### Behavioural Test Battery

A separate cohort of 40 rats (Control *n* = 20, AVD *n = *20) were subjected to a behavioural test battery over a period of 2 weeks, which assessed a range of behavioural domains including locomotion, exploration, anxiety, learned helplessness, active avoidance, sensorimotor gating and nociception. Each test was performed on a separate day, from least to most stressful, in the following order: elevated plus maze (Day 1), holeboard (Day 2), light/dark transition (Day 3), social interaction test (Day 4), forced swim test (Days 8–9), hot plate and tail flick (Day 10), prepulse inhibition (Day 11), active avoidance test. The rats were then tested in the open field test (Day 15). All tests were performed between 8∶00–17∶00h.

Prior to behaviour experimentation, rats were habituated to the testing room for at least 1 h. For the first five tests listed below, a centrally placed camera was used during each trial. Automatic recording of distance travelled was obtained using an image analysis software program (EthoVision XT, Noldus, Wageningen, The Netherlands). A computerised event recorder (Observer 3.1 and XT, Noldus, Wageningen, The Netherlands) was used to manually score behaviours where indicated.

#### Elevated plus-maze (EPM)

The EPM was used to measure anxiety-related behaviour [45]. The EPM was made of opaque grey acrylic and comprised two opposing open arms (10 cm width, 50 cm length) and two closed arms (10 cm width, 50 cm length, 50 cm height), with a square central platform. The maze was raised 100 cm off the ground. Each trial started once the rat was placed on the centre platform facing an open arm and each trial lasted 10 min. The total distance travelled was recorded automatically. The amount of time spent on the open arms, compared to the closed arms, was used as the primary anxiety measure.

#### Holeboard test

The holeboard test allowed measures of exploration as well as locomotion [46]. An opaque grey acrylic insert with four evenly spaced round holes (4 cm diameter) was raised 5 cm from the base of an opaque grey acrylic box (60 cm width, 60 cm length, 45 cm height). Total distance travelled over the 10 min trial was automatically recorded. Analysis of each trial started once the rat had been placed in the centre of the apparatus. Head-dipping, rearing, grooming and inactive behaviours were scored using a computer based event recorder (Observer). Frequency of head-dipping and rearing were the two primary measures of exploration.

#### Light-dark test

The light-dark paradigm allows classification of relative anxiety levels in a rodent [47]. The two-chambered light-dark apparatus consisted of a clear plastic box (46 cm width, 46 cm length, 30 cm height) with a black insert with a small rounded open doorway. The 10 min trial started once a rat was placed through the doorway so that it entered the dark chamber. Frequency of head and body entering the light arena were scored manually using a computer based event recorder (Observer), enabling latency measures to be calculated as the primary measure of anxiety.

#### Social interaction test

The social interaction test can be used to identify abnormal social behaviour [48]. The test measured the behavioural response of two diet- and weight-matched rats. The test was conducted in a circular arena (100 cm diameter, 45 cm height) made of opaque grey acrylic. Two unfamiliar rats were placed on opposite ends of the arena with each pair treated as the unit of observation (i.e. *n* of 1 = 1 pair of rats, thus Control *n* = 10, AVD *n* = 10). Social behaviour was scored using a computer based event recorder (Observer) with frequency and latency of investigation behaviour being the primary measure of social interaction. Investigation behaviours included sniffing, crawling over and under, allogrooming, following, and aggressive behaviour (biting, chasing, boxing). Other behaviours recorded included rearing, self-grooming and aggressive behaviour.

#### Two-day forced swim test

The forced swim test provided a measure of learned helplessness [49]. Rats were placed in a round dark blue container (35 cm diameter, 80 cm height) filled to 30 cm with water maintained at 25–28°C, from which escape was not possible. After the 10 min test period, the rat was removed from the container, wrapped in towel and put in a warm area to dry for 30 min. The following day, the rat underwent the same procedure for 5 min. Pairs of rats were tested consecutively and the water was changed between every second rat. The amount of time the rat spent immobile, mobile and actively diving were recorded manually using a computer based event recorder (Observer). Time spent immobile on the second day was the primary measure of learned helplessness.

#### Nociception: Tail flick and hot-plate test

The tail flick test measured thermal pain sensitivity by assessing spinal nociception [50]. Rats were tested using a tail flick analgesia meter (Harvard Apparatus, Ltd., Kent, England), by aligning the rat’s tail with a beam of light while the rat was restrained with a small towel. The light bulb (150W at 30% intensity) was manually switched on and the built-in timer automatically switched off after the tail moved away from the warm stimulus. Each trial was repeated three times with a minimum interval of 30 s between trials.

The rats were tested on the hot-plate to measure pain threshold [51]. The rat was placed onto a 53°C hot plate (Harvard Apparatus, Ltd., Kent, England) surrounded by a clear Perspex cylinder (20 cm diameter, 27 cm height). The test ran for a maximum time period of 30 s or until an endpoint was observed (latency to lick the hind paw).

#### Prepulse inhibition (PPI) of acoustic startle response (ASR)

The normal response from a loud sound can be attenuated through a weaker prestimulus, also referred to as sensorimotor gating [52]. A disruption in PPI demonstrates a failure to filter out extraneous stimuli and is related to abnormal sensorimotor gating [52]. The current PPI paradigm was based on a previous method described [53]. Additional rats were tested for PPI responses (Control *n = *34, AVD *n = *35).

Rats were placed in a Plexiglass cylinder (8.7 cm diameter, 20 cm length) mounted on an elevated Plexiglass base within a ventilated, dark enclosure (SR-LAB, CA, USA). Background noise was set to 70 dB and acoustic pulses of white noise were delivered through a speaker 20 cm above the cylinder. Testing sessions began with a 300 s acclimation period with 70 dB background noise, followed by five 110 dB startling pulses 20 s apart. A total of 120 trials (five of each trial type) were then presented in a pseudorandom order with an average inter-trial interval of 15 s (range 10–20 s). Prepulse intensities of 74, 78 or 86 dB were presented with prepulse-pulse intervals of 8, 16, 32, 64, 128 or 256 ms, totalling 18 different trial combinations. Single pulses of 70, 80, 90, 100, 110 and 120 dB were also presented throughout the session to assess ASR. Testing ended with five 110 dB startling pulses 20 s apart, to allow calculation of within-session habituation. Startle responses from each trial were determined by mean amplitude, measured by an accelerometer. The median values from each trial type were used for analysis. Percentage PPI was calculated as: [(startle amplitude of ASR trial - startle amplitude on prepulse trial)/startle amplitude of ASR trial]×100.

#### Active Avoidance

The active avoidance test allowed rats to learn a conditioned avoidance response [54]. An active avoidance protocol previously described was modified and adopted [15]. The active avoidance chamber (Gemini Avoidance System, San Diego Instruments, SD USA) had two compartments (21 cm width, 26 cm length, 18 cm height) with a stainless steel gate (7 cm width, 10 cm height) connecting the two compartments together. Two active avoidance chambers were run in parallel. An acclimation period of 300 s in the left hand compartment preceded 80 trials. Each trial lasted 20 s and comprised of a 4 s conditioned stimulus (CS) and then initiation of a 16 s unconditioned stimulus (US). The CS was a cue light (15 W bulb) and tone and the US was a 0.6****mA shock delivered to the stainless steel grid floor. Between each trial was an inter-trial interval where no stimulus was presented for 20 s. The gate was closed during the inter-trial interval, but open during the presentation of the CS and US. The learned response was to move from one compartment, at which time the shock ended. Eight infrared photobeams in the compartments allowed detection of animal location. The Gemini software automatically recorded the latency for the animal to respond and classified each response as escape (response <4 s), avoidance (response <20 s) and no response (response = 20 s). The percentage of conditioned avoidance responses (%CAR) was the primary measure of avoidance learning.

#### Open field test

Rats were placed in an activity monitor box. Vertical and horizontal activity was automatically measured by beam breaks (three 16 beam infrared arrays) and analysed using an activity monitor program. Horizontal activity and vertical counts were the main measures of locomotion and exploration.

### Behavioural Pharmacology

#### Pharmacological agents

D-amphetamine and MK-801 purchased from Sigma (MO, USA) were used to assess psychomimetic-induced hyperlocomotion. Sodium chloride was obtained from AstraZeneca (NSW, Australia). D-amphetamine was made up in saline solution at 1.25****mg/mL and MK-801 at 0.5****mg/mL. Drug stock solutions were stored at −20°C in 2****mL aliquots and warmed to room temperature on the respective testing day. Sodium chloride (0.9%), amphetamine (1.25****mg/kg) and MK-801 (0.5****mg/kg) injections were administered intraperitoneally (IP) at 1****mL/kg.

#### Procedure

Behaviourally-naïve rats were habituated to the testing room 1 h prior to experimentation (Control *n = *34, AVD *n = *35). Rats were placed in a clear box as described previously (see open field test) with a layer of aspen bedding (P.J. Murphy Forest Products Corp., NJ, USA). Four activity chambers were run in parallel. The rats were habituated to the test chambers for 30 min and then given a saline injection at T30-T32 and monitored for another 30 min. At T60-T62 rats were given a amphetamine injection and monitored for a final 120 min. The following day, the rats (Control *n = *21, AVD *n = *21) were tested again using exactly the same protocol as day one, but an MK-801 injection was given instead of amphetamine. Horizontal activity (psychomimetic-induced locomotion) was the primary measure of locomotion.

### Operant Behaviours

Training and testing was conducted in a bank of eight operant chambers, which were individually housed in sound-attenuating ventilated cubicles (Med-Associates, VT, USA). Each chamber consisted of five square apertures (2.0 cm in diameter), equally spaced horizontally across a curved aluminium front wall, 2.0 cm above a stainless steel grid floor (Fig. 2, 3). A yellow light-emitting diode (LED) stimulus light (6.4 mm in diameter) was located at the back of each aperture, with an infrared detector 1.0 cm from the front of the aperture. On the back wall was a food magazine (5.0 cm in diameter) connected to a 45 mg pellet dispenser. A light was located inside the magazine (1.0 cm in diameter) and an infrared detector situated horizontally across the magazine. The house light was located on the ceiling above the magazine. Two retractable stainless steel levers (5.0 cm; not used in 5CSRTT or 5CCPT but see DMTS protocol) were located either side of the food magazine, 6.0 cm above the steel grid floor. Each lever was accompanied by a corresponding cue light (2.5 cm in diameter), located 6.5 cm above the lever. The sidewalls and ceiling were made of clear polycarbonate and an infrared camera was installed above each chamber to monitor performance. All responses were detected by infrared beam breaks and SmartCtrl Package 8-In/16-Out managed control of stimuli and response recording, with additional interfacing by MED-PC for Windows (Med Associates Inc., VT, USA).

#### 5C-SRT training protocol

Behaviourally naïve rats (Control *n = *8, AVD *n = *8) were trained to perform the 5C-SRT based on a published protocol [16], [55]. Over the first five training days rats learnt to retrieve sugar pellets (45 mg; dustless precision sugar pellets, Able scientific, WA, Australia) from the food magazine, before being trained to detect a stimulus light at one of five locations. Rats progressed through several stages until they reached criteria for 5C-SRT. Each session consisted of 100 trials or terminated after 30 min, whichever came first. Various parameters were gradually adjusted across sessions including duration of the stimulus (30, 20, 10, 5. 2.5, 1.25, 1 s), limited hold period (30, 20, 10, 5 s) and reward duration (10, 5, 2 s). The rodents were transported to the behaviour testing room 30 min before testing commenced to acclimatize to the dimly lit. A rat was then placed in an operant chamber and the session was initiated with the delivery of a food pellet to the lit food magazine. Retrieval of the food pellet started a fixed inter-trial interval (ITI) of 5 s, during which all lights were turned off. At the end of the ITI, a 1 s (stimulus duration) light stimulus was presented in one of the five light apertures opposite the food magazine. Rats had up to 5 s (limited hold) to make a correct response (Hit) by nose-poking the aperture that was illuminated. This resulted in delivery of one food pellet, accompanied by the food magazine light, which remained on for 2 s (reward duration). A head entry into the food magazine broke an infrared beam and was recorded. A nose-poke in any of the remaining apertures was deemed an incorrect response (Miss) and resulted in a 5 s time-out during which the house light illuminated. Failure to respond at all during the limited hold was an omission error, which also resulted in a 5 s time-out. Responses occurring before stimulus presentation (during the ITI) were termed premature responses, resulting in a 5 s time-out. Additional responses during this time-out period were also considered premature responses, however the duration of the time-out was unaffected. Nose-pokes to any aperture after the first nose poke were recorded as perseverative responses. Each rat was always tested in the same operant chamber across sessions.

During the final phase of training (variable ITI protocol) the number of trials was increased to 120 and each trial was separated by a variable ITI of 3, 4, 5, 6 or 7 s. Rats were considered to have acquired the task when they made greater than 60 correct responses and fewer than 20 errors of omission over three consecutive days.

Performance measures consisted of: choice accuracy (calculated as [number of correct responses/(number of correct+number of incorrect responses)], correct and incorrect response latency (time taken to respond correctly or incorrectly after the stimulus presentation), omissions, premature responses, perseverative responses, head entries to the food magazine and reward latency (time taken to collect the food pellet from the food magazine after a correct response).

#### 5C-CPT

Sessions in the 5C-CPT task consisted of 120 trials or terminated after 30 min. Eighty trials were target trials as just described for 5C-SRT, where the cue stimulus would appear in one of the five apertures. Forty non-target trials were also randomly interspersed to further assess impulsivity. On a non-target trial all five apertures were illuminated, and the rodent had to withhold from responding to receive a food reward. Consistent with human continuous performance tasks, successful inhibition of a response in a no-go trial was termed a correct rejection (CR) and a response in a no-go trial was recorded as a false alarm (FA), which incurred a time-out. Subjects were required to be on the 5C-CPT for a minimum of 10 days. On target trials we recorded accuracy, the proportion of hits (P(Hit) = Hit/Hit+Miss), omission rate, number of premature responses, number of perseverative responses as well as the latencies to make a correct response, an incorrect response and to collect reward were used to analyse the performance of rats. One-third of the trials for the 5C-CPT study, were non-target trials and therefore for this study the following variables were added to the analysis; proportion of false alarms (P(FA) = FA/FA+CR), number of perseverative FA, latency to make a FA response and latency to retrieve a reward for a correctly withheld response.

#### DMTS

Behaviourally naïve rats (Control n = 8, AVD n = 8) were trained in the same operant chambers as described previously on the DMTS task based on previously published protocols [56]. In the initial training phase rats learnt to retrieve sugar pellets from the illuminated food magazine. Next they were trained to respond to any lever when both were presented in the chamber. Behaviour was further shaped with food reward and the rats progressed through different stages until they reached baseline criteria for DMTS. The DMTS trial commenced with one of the two retractable levers (randomly selected) being presented in the chamber, accompanied by the corresponding cue light. This was defined as the sample phase and the rat was required to press the lever. The lever was then retracted and the rat had to nose-poke the illuminated food magazine. After a random variable delay phase (2, 4, 8, 12, 18, 24 s), both levers were extended back into the chamber for the test phase. The correct response was to ‘match to sample’ and the rat was required to push the same lever that was extended in the sample stage, to which a food pellet was dispensed and the food magazine lit up. An incorrect response to the other, ‘non-match to sample’ lever resulted in a time out, during which the house light was illuminated. After a 5 s ITI the next trial commenced.

### Brain Neurochemistry

#### Tissue collection

Twenty-week-old drug and behaviourally naïve rats (10-weeks on AVD-deficient or control diet) received an overdose of pentobarbitone (Lethabarb, Virbac, NSW, Australia), before the brain was rapidly dissected and weighed. The brain was then placed in 10 mL of ice-cold sodium chloride solution (0.9%) for 5 min. A brain block on ice was utilised to cut coronal slices (1.4 mm wide) and the PFC and striatum were dissected. Tissue was frozen on dry ice and stored at −80°C.

#### High performance liquid chromatography and analysis

Catecholamines and amino acids from brain tissue were measured by high performance liquid chromatography with electrochemical detection for catecholamines [12] and fluorescent detection for amino acids [57]. Brains were thawed on ice, weighed (wet weight) and diluted based on weight with a minimum volume of 0.1****mL of 0.1****M perchloric acid and 50****ng/mL deoxyepinephrine (internal standard for determination of catecholamines). They were then homogenised on ice using probe sonication (5 s at 60% amplitude; Vibra-Cell, Sonics & Materials, CT, USA) and centrifuged at 13,000 rpm for 5 min at 4°C with the supernatant filtered by a 0.22 µM nylon syringe filter (PM Separations, QLD, Australia). Ten microlitres of sample was injected into the HPLC system, which consisted of a degasser, autosampler and an isocratic HPLC pump (Model 1100, Agilent Technologies, CA, USA), a Sunfire C18 column, 4.6 mm×150 mm, 5 um; (Waters Corporation, MA, USA) and a Coulochem III (ESA Laboratories, MA, USA) electrochemical detector. The mobile phase consisted of a 12% acetonitrile/75 mM potassium dihydrogen phosphate buffer containing 25 uM EDTA and 1.7 mM octane sulfonic acid adjusted to pH 4.13 with phosphoric acid. Flow rate was 1.2 ml/min. Detector settings were as follows: - conditioning cell (Model 5020, ESA Laboratories, MA, USA) at +350 mV; analytical cell (Model 5014B, ESA Laboratories, MA, USA) with the first and second electrodes maintained at −150 and +250 mV, respectively. Amino acids were analysed by HPLC using pre-column derivatisation and fluorescence detection. Samples were maintained at 4°C and the derivatisation protocol was conducted by the autosampler as follows: −10 µL of 1 nM/µL homoserine (internal standard) was mixed with 10 µL of sample; then 100 µL of diluent (methanol/water, 50∶50, v/v) was added and mixed; 2 µL of borate buffer (Agilent Technologies, CA, USA) was drawn into the loop; then 1 µL was drawn from the sample and mixed in the loop; finally 0.5 µL of OPA reagent (Agilent Technologies, CA, USA) was drawn and mixed in the loop which was then injected into the system after 1 min. The system consisted of an Agilent 1200 degasser, binary pump, autosampler with thermostat and fluorescence detector (Model 1200, Agilent Technologies, CA, USA) equipped with a Phenomenex Gemini C18, 4.6 mm×150 mm, 3 um column (Phenomenex, CA, USA). The mobile phase consisted of 0.05 M sodium acetate, tetrahydrofuran and methanol (50∶1:49, v/v) adjusted to pH 4.0 using 100% acetic acid. Flow rate was 0.75 ml/min with a 40 min run time and the fluorescence detector was set to an excitation wavelength of 337 nm and an emission wavelength of 454 nm. Data was stored and processed with ChemStation software (B1.03.02, Agilent Technologies, Inc.; CA, USA) and integrated independently by two people. Compound identity was determined using retention time, and analyte amount was quantified relative to the internal standard area and calibrated using standard curves. Sample dilution was adjusted and presented as nanogram per gram (ng/g) wet tissue. Ratios of these were used to assess conversion of neurotransmitters and metabolites.

### Statistical Analysis

Results were analysed using the SPSS (ver. 17, SPSS Inc., Chicago, Illinois) software package. Several different analyses were performed, including independent samples t-tests with Levene’s test for equality of variance, to examine the effect of diet. Repeated measures ANOVAs were performed on some of the behavioural data; the open field test, ASR and PPI and the variable ITI contingency. Non-parametric tests (for example, Mann-Whitney U) were used when variances were non-homogenous. A repeated measures ANOVA was performed to examine between-subjects effects of neurotransmitter and metabolite level in all brain regions. Significant effects were followed up post hoc with independent samples t-tests. Values of *p*<0.05 were considered to be significant. All data are reported as mean ± standard error of the mean (S.E.M.). Values with *p*<0.05 indicated statistical significance.
